# Pulmonary Function Responses During Inspiratory Muscle Training in Patients with Chronic Heart Failure: A Systematic Review and Meta-Analysis of Randomized Controlled Trials

**DOI:** 10.3390/healthcare14172692

**Published:** 2026-08-24

**Authors:** Georgios Mitsiou, Irini Patsaki, Afrodite Evangelodimou, Eirini Grammatopoulou

**Affiliations:** Laboratory of Advanced Physiotherapy, Department of Physiotherapy, University of West Attica, 12243 Egaleo, Greece; gmitsiou@uniwa.gr (G.M.); aevangelodimou@uniwa.gr (A.E.);

**Keywords:** inspiratory muscle training, maximum inspiratory pressure, diaphragm thickness, dyspnea, functional vital capacity, maximum oxygen uptake

## Abstract

**Background:** Exercise intolerance is a common symptom in patients with chronic heart failure (CHF) and is often associated with reduced pulmonary function. Breathing exercise, including inspiratory muscle training (IMT) has been shown to alleviate respiratory symptoms in patients with pulmonary and cardiac disease. The purpose of this systematic review is to assess the impact of IMT on maximal inspiratory pressure (Pimax), diaphragm thickness, functional vital capacity (FVC), dyspnea and maximum oxygen uptake (VO_2peak)_ in patients with CHF. **Methods:** Database research was conducted (PubMed, EMBASE, Cochrane Library of Controlled Trials) by using an appropriate algorithm to indicate the IMT effect on pulmonary function studies. Eligibility criteria included pulmonary function measurements following IMT in patients with CHF. A continuous random effect model (PROSPERO-CRD420261360295) was used to calculate standard mean differences (Std.MD) in all outcomes (between an experimental and control group). **Results:** A total of 395 participants (13 randomized controlled trials) were identified. More studies indicate improvement in Pimax after IMT (Pimax: Std.MD: 1.07; 95%CI, 0.72–1.42; *p* < 0.00001). Functional vital capacity (Std.MD, 0.41; 95%CI, 0.03–0.79; *p* = 0.03) and dyspnea (Std.MD, 1.11; 95%CI, 0.57–1.66; *p* < 0.0001) were also improved. Moreover, VO_2peak_ assessed by cardiopulmonary exercise testing before and after IMT showed statistical significance (VO_2peak_: Std.MD: 0.86; 95%CI, 0.06–1.66; *p* = 0.04) in trained patients with CHF compared to controls. **Conclusions:** The meta-analysis indicates that isolated IMT performed either as short- to medium-term intervention (4 to 8 weeks) with moderate to high intensity (40 to 90% Pimax) or long-term program (8 to 12 weeks) with low intensity (30% Pimax) promotes pulmonary function and metabolic responses. More studies are required to examine the impact of IMT on diaphragm thickness in patients with chronic heart failure.

## 1. Introduction

Heart failure is a clinical syndrome that reflects anatomical and biological disorders of the myocardium and peripheral organs [1] and constitutes the leading cause of mortality in patients with cardiovascular disease [2]. Exercise intolerance and reduced functional capacity are common symptoms in patients with chronic heart failure (CHF) and are often associated with pulmonary dysfunction [3]. In particular, the reduced ventilation and cardiac output [4] affect diaphragm function [5], lead to respiratory muscle weakness [6] and are related to poor prognosis and impaired clinical outcomes in patients with CHF [7,8]. Studies on anatomical aspects of the diaphragm have indicated diaphragm thickness as a marker of respiratory muscle dysfunction. Indeed, diaphragmatic atrophy provokes a reduction in respiratory muscle strength and is associated with reduced exercise capacity in patients with CHF [9].

Cardiac rehabilitation is used as standard care for patients with CHF to improve cardiovascular function and skeletal muscle strength [10]. Aerobic exercise training is recommended as a fundamental modality to improve cardiac function and functional capacity [11]. Breathing exercise such as inspiratory muscle training (IMT) has been shown to reduce dyspnea and alleviate respiratory symptoms in many diseased populations [12,13], including patients with cardiovascular disease. Several studies have examined the effects of IMT on respiratory muscle strength and pulmonary function in CHF; however, in most of them, IMT was performed in combination with aerobic and resistance exercise as part of a comprehensive rehabilitation program [14,15]. Winkellman and colleagues [16] have shown that adding IMT to cardiac rehabilitation has a significant effect on respiratory function and overall exercise capacity. Thus, IMT constitutes an effective complementary treatment to aerobic and resistance exercise that might provide additional benefits to reverse respiratory muscle weakness. Indeed, Vibarel et al. [17] found that aerobic exercise training performed without IMT did not significantly increase inspiratory muscle strength. However, whether isolated IMT might improve respiratory muscle strength and pulmonary function in patients with CHF needs to be clarified. Thus, further research is required to examine whether combining IMT with standard interventions provides additional benefits beyond either intervention alone and to determine the impact of each exercise modality on pulmonary function.

Previous systematic reviews that have examined the effect of IMT on CHF have mainly focused on the impact of IMT on functional capacity and quality of life [18,19] and have observed significant changes on these outcomes. These reviews have also included studies conducted up to 2019. They have mainly compared the results of randomized trials and examined the effects of IMT in combination with other interventions [19]. Some studies that have also examined the effects of isolated IMT on pulmonary function were primarily designed as controlled trials [20,21], increasing methodological heterogeneity in the available evidence [18]. Although respiratory retraining appears to be effective in managing patients with CHF, to date, the effect of IMT on pulmonary function and dyspnea in these patients has not been elucidated thoroughly. Furthermore, whether isolated IMT performed during short-term or long-term training programs can improve diaphragm thickness and exercise capacity in CHF needs to be clarified further. The purpose of this systematic review is to assess the impact of IMT on maximum inspiratory pressure (Pimax), diaphragm thickness, functional vital capacity (FVC), dyspnea, and maximum oxygen uptake (VO_2peak_) in patients with CHF.

## 2. Methods

### 2.1. Data Sources and Literature Search

This systematic review and meta-analysis on the effect of IMT on pulmonary function in patients with CHF was conducted in compliance with the Preferred Reporting Items for Systematic Reviews and Meta-Analysis (PRISMA) guidelines [22]. The protocol was registered in PROSPERO (Registration ID: CRD420261360295). A thorough literature search was conducted in PubMed, Embase and Cochrane Library for relevant articles. Studies already published on the subject were considered for inclusion irrespective of publication year. The initial search was conducted in December 2025. Weekly alerts were also received and updated up until June 2026.

### 2.2. Selection Criteria

The inclusion criteria included studies that reported the effect of IMT on pulmonary function and exercise capacity in patients with CHF irrespective of sex, were peer-reviewed and published in English, were RCTs, reported the pre-and post-intervention measures, and included only adult subjects ≥18 years of age. Studies that included patients with either reduced or preserved ejection fraction, including those with heart failure and accompanied by respiratory muscle weakness, were also included. Concomitantly, eligible studies met at least one of the following criteria: (a) measurement of Pimax, diaphragm thickness, FVC, dyspnea and VO_2peak_; (b) use of IMT intervention, (c) inclusion of patients with CHF; and (d) comparison with control groups, placebo, or another intervention for the treatment of patients with heart failure. Studies meeting these criteria were included in the analysis. In addition, IMT was defined to allow incentive (based on volume) or threshold (based on resistance) training. Although these modalities may differ in the training stimulus and physiological mechanisms, both aim to improve pulmonary function. Taking into consideration that the ideal protocol for evaluating the real effectiveness of the IMT has still not been established [23], the two approaches were analyzed together to estimate the overall effect of IMT.

To identify the impact of isolated IMT, studies were excluded when IMT was performed as part of a multimodal exercise intervention. Combined interventions can affect pulmonary function and are considered as the standard care for patients with CHF [10]. However, to distinguish the effects of isolated IMT from those of conventional exercise training as well as to minimize potential confounding, studies that used combined interventions were excluded. Other literature (systematic reviews, meta-analysis, prospective studies, book chapters, conferences, case reports, posters) was also excluded.

Two reviewers (G.M. and A.E.) independently searched for eligible publications in selected clinical journals, and reviews, as well as in the reference lists of papers suitable to be included in our systematic review. Full-text articles of potentially eligible studies were then independently assessed by the same reviewers. Conflicts between them were resolved by a third investigator (E.G.).

### 2.3. Data Extraction Studies

Eligible studies were considered randomized controlled trials (RCTs), and the objective measurements of outcomes included absolute values or % changes. Data were extracted on (i) author and year, (ii) anthropometric characteristics (i.e., age, gender), (iii) duration and intensity of IMT, (iv) timing of measurements. The 95% confidence interval (CI) was reported, or the data were presented such that the 95% CI could be calculated. In addition, for two studies [24,25], standard errors were converted into standard deviations (SDs) using the equation: SD = SE Χ √n [26]. In two studies [24,27], where data were not provided in the main texts or tables, they were extracted from the relevant figures using the WebPlotDigitizer (version 3.x) [28]. Finally, for one eligible study [29], the estimation of sample mean ± SD proposed by Wan et al. [30] was used to convert the data reported in the median and range.

### 2.4. Statistical Analysis

Revman 5.4.1, 2020 software (The Cochrane Collaboration, Oxford, UK) was used for quantitative analysis [31]. Random-effects models were adopted to account for heterogeneity due to the differences in study populations, IMT interventions, and to calculate the mean differences in the outcomes between interventions and control groups. Heterogeneity was explored qualitatively and quantitatively using the chi-square test and I^2^ statistic [32] (range: absolute homogeneity 0% to 100%, highest heterogeneity). Since different scales were adopted in the eligible studies, we used SMDs instead of absolute mean differences to standardize the findings to a uniform scale [33] and to account for deviations in the units of measurement. The post-trial values for outcomes between the intervention and control groups were used for comparisons to allow direct estimation of the between-group difference to prevent potential confounding and to minimize bias. Post-trial values were analyzed using an Analysis of Covariance (ANCOVA) model, with group assignment as the fixed factor and baseline values included as a continuous covariate. In one case [34], where the baseline values differed significantly between the intervention and control groups, we compared pre/post values for the intervention group. The level of significance for changes in the outcome measures was set at *p* ≤ 0.05.

### 2.5. Risk-of-Bias Assessment

During the selection process, we accepted only RCTs to provide more robust estimates on the effect of IMT; controlled trials without randomization, single-group studies and cross-sectional studies were excluded. For the eligible RCTs, the updated Risk of Bias 1 Cochrane library tool (the “Cochrane Collaboration’s tool” [33]) was adopted to measure and report a score for each included study regarding the quality of the reported results. Two authors (G.M. and Ε.Μ.) independently assessed the risk of bias in the included studies; another author (I.P.) acted as a referee to resolve any disagreement.

The certainty of evidence for the main outcomes was assessed using the GRADE (Grading of Recommendations Assessment, Development and Evaluation) framework [35] based on five domains: risk of bias, inconsistency, indirectness, imprecision, and publication bias. Each outcome was rated as high, moderate, or low. The GRADE results after evaluation are presented in the Results Section.

## 3. Results

### 3.1. Searching Procedure Results

**Figure 1:**
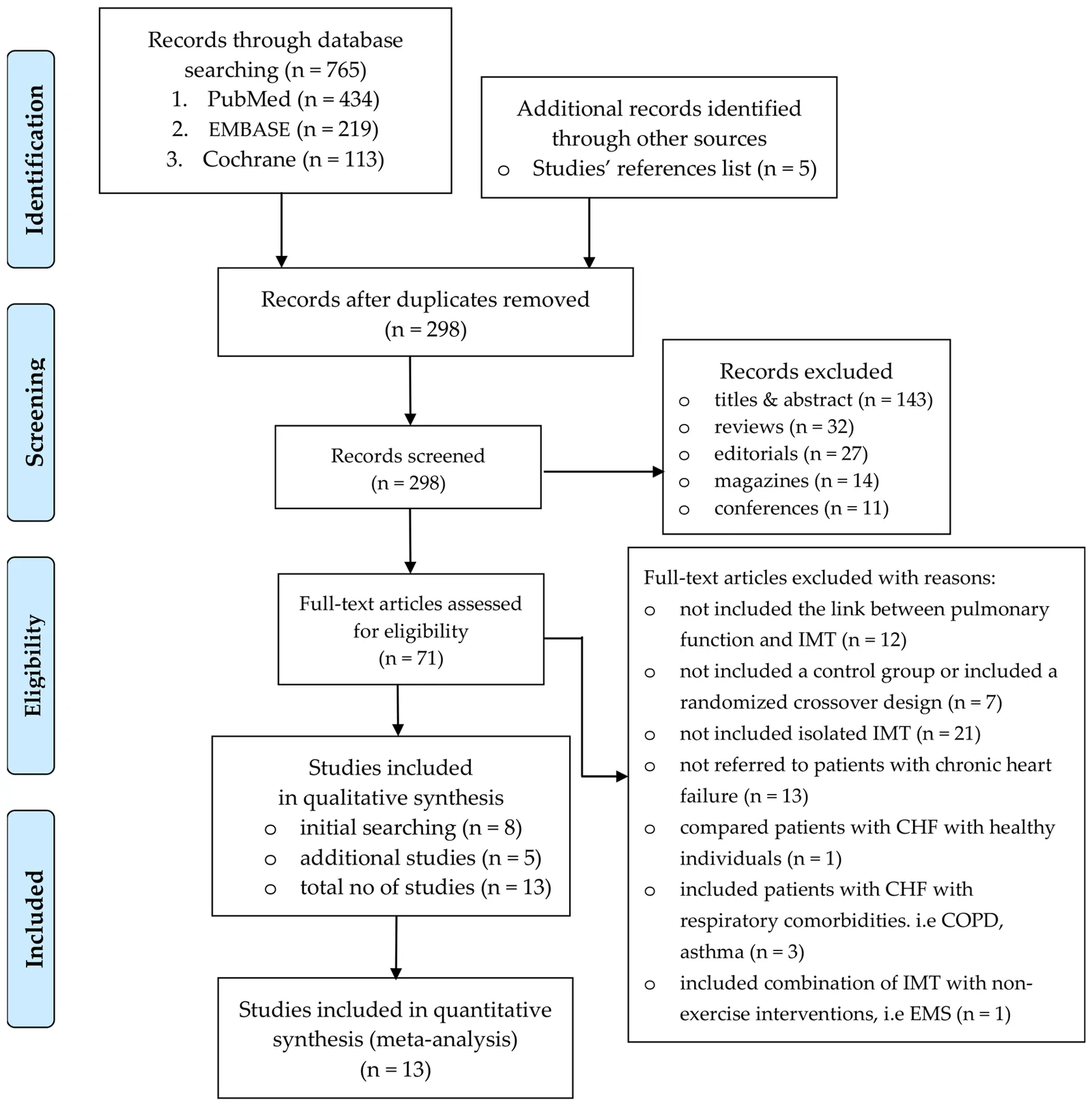
PRISMA flow chart of the study. CHF: chronic heart failure; COPD: chronic obstructive pulmonary disease; EMS: electrical muscle stimulation. IMT: inspiratory muscle training.

The studies included in this systematic review were published between 1999 and 2023. Eight RCTs [24,25,27,29,36,37,38,39] met the inclusion criteria (Figure 1). Five additional studies [34,40,41,42,43] were added through secondary manual searching of the reference list of the eligible studies. Details of full-text articles that were reviewed but excluded for different reasons are provided in Figure 1.

### 3.2. Characteristics of the Included Studies

Characteristics and results of the included studies can be found in Table 1. Two studies included patients with congestive heart failure [24,39], two included patients with heart failure and inspiratory muscle weakness [38,40], one study included patients with preserved injection fraction [29], and one study was conducted IMT in older patients with heart failure. Seven studies [25,34,36,37,39,41,42,43] included patients with heart failure with reduced ejection fraction. Furthermore, nine studies adopted IMT with the use of a threshold, and four used an incentive method. Three studies [34,36,42] used short-term IMT programs, two used medium-term IMT programs (8 weeks) [37,41], and eight studies used long-term IMT (10 to 12 weeks) [24,25,27,29,38,39,40,43]. Overall, the included studies in the current systematic review involved 395 patients with CHF, 199 participated in the intervention groups and 196 were included in placebo or control groups.

### 3.3. Risk of Bias and Assessment Results

A summary of the risk-of-bias assessment is presented for the included RCTs. A detailed description of the risk-of-bias assessment for all the eligible studies in the current systematic review is presented in Table 2 (Supplementary File, Figure S1). The CONSORT (Consolidated Standards of Reporting Trials) [44] 25-item checklist was used to measure and report a score for each RCT regarding the quality of the reported results. The process was independently performed by two reviewers (G.M. and I.P.; disagreements were resolved by E.M.). The evaluation of the reporting data showed a mean score of 17.7 out of 25 for the RCTs (Table 3). The score represents the number of items on the checklist that were reported satisfactorily in each study (a high or low score represents high or low adherence to reporting guidelines).

### 3.4. Reporting of the Outcomes

#### 3.4.1. Impact of IMT on Pulmonary Function in Patients with CHF

**Figure 2:**
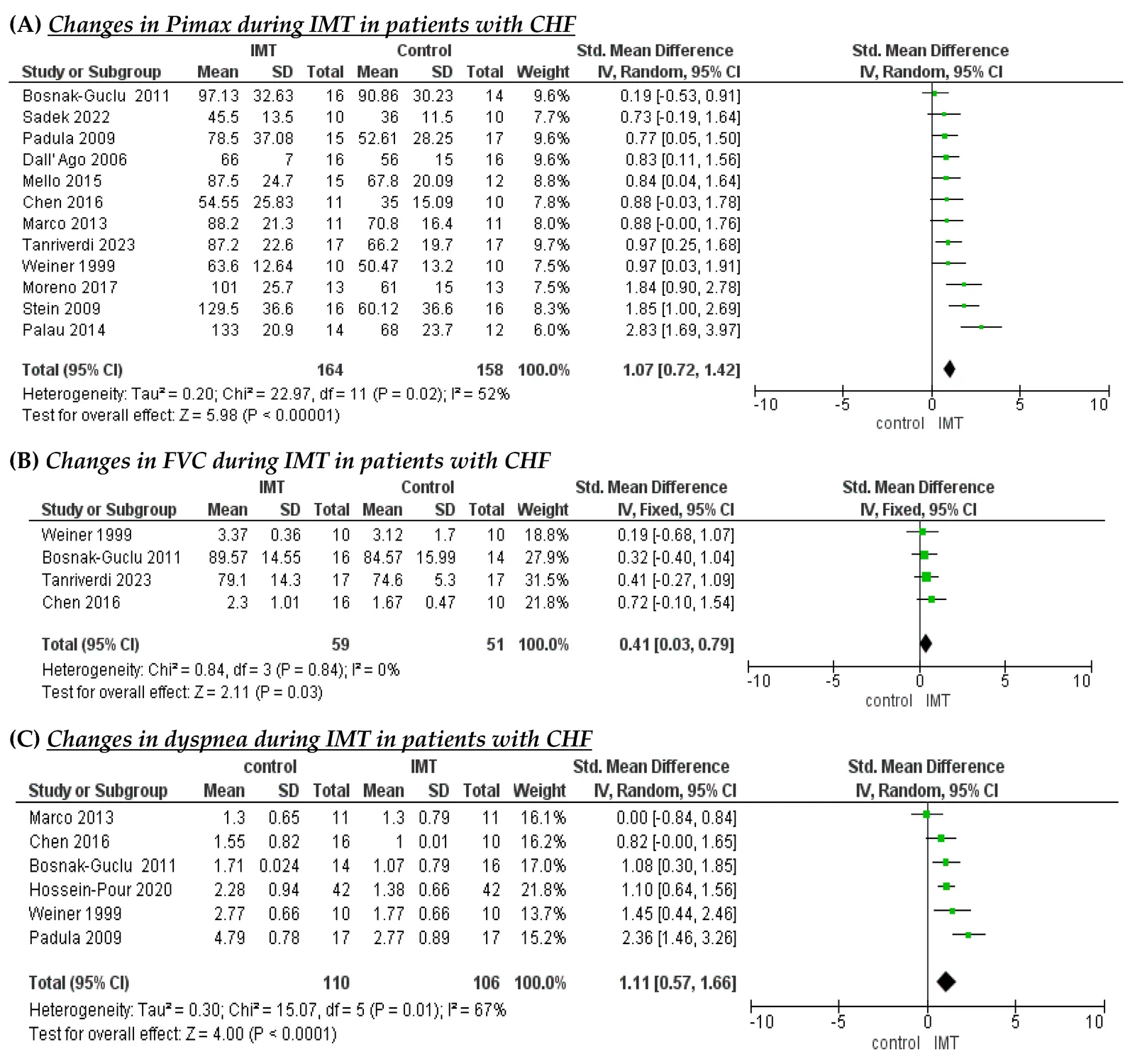

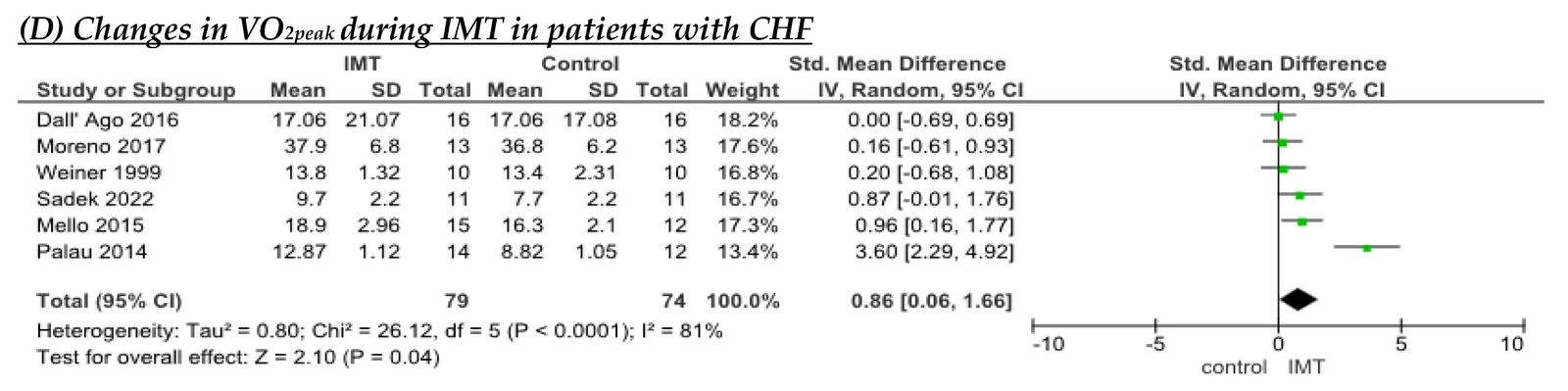
Pulmonary and metabolic responses during IMT in patients with CHF [24,25,27,29,34,36,37,38,39,40,41,42,43].

We used a random-effects model because after analyzing the data in the IMT and control groups, heterogeneity was found in some analyses. The majority of the studies (12) evaluated the effect of isolated IMT on Pimax in patients with CHF compared to controls following either short-term, medium-term, or long-term training programs. Most of the studies clearly indicate an increase in respiratory muscle strength and significant Pimax changes following IMT (Std.MD: 1.07; 95%CI, 0.72–1.42; *p* < 0.00001, Figure 2A). Four studies assessed pulmonary function by evaluating FVC during medium- and long-term exercise training and revealed a significant increase after IMT (Std.MD, 0.41; 95%CI, 0.03–0.79; *p* = 0.03, Figure 2B). In addition, six studies found that dyspnea was improved in patients with CHF following IMT rehabilitation (Std.MD: 1.11; 95%CI, 0.57–1.66; *p* < 0.0001, Figure 2C). Results were similar when dyspnea was assessed either with the MRC score or with the Borg scale. Moreover, in six studies, maximum oxygen uptake assessed by cardiopulmonary exercise testing before and after IMT showed statistical significance in patients with CHF compared to controls (Std.MD: 0.86; 95%CI, 0.06–1.66; *p* = 0.04, Figure 2D). Finally, the funnel plot assessment using Egger’s test indicated no publication bias.

#### 3.4.2. Evidence of Effectiveness (Grade Analysis)

Due to the inclusion of various protocols of IMT, moderate to high heterogeneity was observed in some meta-analyses. The evaluation of quality of evidence (Table 4) for patients with CHF via GRADE (Grading of Recommendations Assessment, Development, and Evaluation) analysis for Pimax and FVC showed high quality of evidence. The analysis of dyspnea and VO_2peak_ showed moderate quality of evidence.

### 3.5. Qualitative Perspective

A limited number of studies assessed the primary outcome (diaphragm thickness) during IMT in patients with CHF and found contradictory results. In a recent study, 8 weeks of IMT performed with incremental intensity (30–70% Pimax) increased diaphragm thickness at end expiration [37]. This increase seems to be attributed to the inspiratory muscle weakness observed in the included patients with CHF and the delayed diaphragmatic fatigue. This study also found a parallel increase in inspiratory muscle strength after IMT. In a second study [45], although different results of diaphragm dimensions in expiration and residual volume were reported, changes in diaphragm thickness were not observed after short-term IMT (4 weeks) performed up to 60% Pimax. The contradictive results in the latter study may be attributed to differences in IMT duration and training intensity progression of patients with CHF. Both studies determined the thickness of the diaphragm with the use of ultrasound and performed IMT with increased progressive intensity. However, physiological changes were observed only when IMT was performed over a longer period (8 weeks), suggesting it provided sufficient stimulus. The shorter intervention (4 weeks) was insufficient to induce changes in anatomical aspects of the diaphragm. Moreover, variations in measurements and sample size may have contributed to inconsistent findings in the two studies. Therefore, while IMT appears to enhance inspiratory muscle performance in patients with CHF, its impact on diaphragm morphology remains unclear and may depend on training dose and patient characteristics. However, the latter controlled trial compared patients with CHF to healthy individuals; thus, it was excluded from the eligible studies and analysis was not performed.

## 4. Discussion

The present systematic review and meta-analysis examined the effects of IMT on Pimax, FVC, dyspnea and VO_2peak_ in patients with CHF. The results indicate that isolated IMT led to an increase in inspiratory muscle strength and VO_2peak_ and improvement in FVC and dyspnea. The eligible studies indicate IMT intervention using different training loads in terms of duration and intensity affects the outcomes. Indeed, when training loads of 30% Pimax were used with longer duration or when IMT was performed with moderate (40–60% Pimax) to high intensity, reaching up to 90% Pimax, even after short- or medium-term intervention, benefits in pulmonary and metabolic parameters were observed. Moreover, recent data indicates that high-intensity interval-based IMT, performed three times per week at the highest tolerable inspiratory resistance (i.e. 60 to 70% Pimax) seems to promote pulmonary and metabolic responses in patients with CHF and better adherence to the intervention [36,37]. The restricted findings reported on changes in diaphragm thickness remain preliminary as the sparsity of data precluded a quantitative meta-analysis. The current evidence is insufficient to determine whether IMT consistently induces structural adaptations of the diaphragm in patients with CHF. Therefore, such evidence should be interpreted with caution, and conclusions regarding physiological changes in anatomical structures of the diaphragm should remain tentative.

### 4.1. Responses of IMT to Pimax

**Figure 3:**
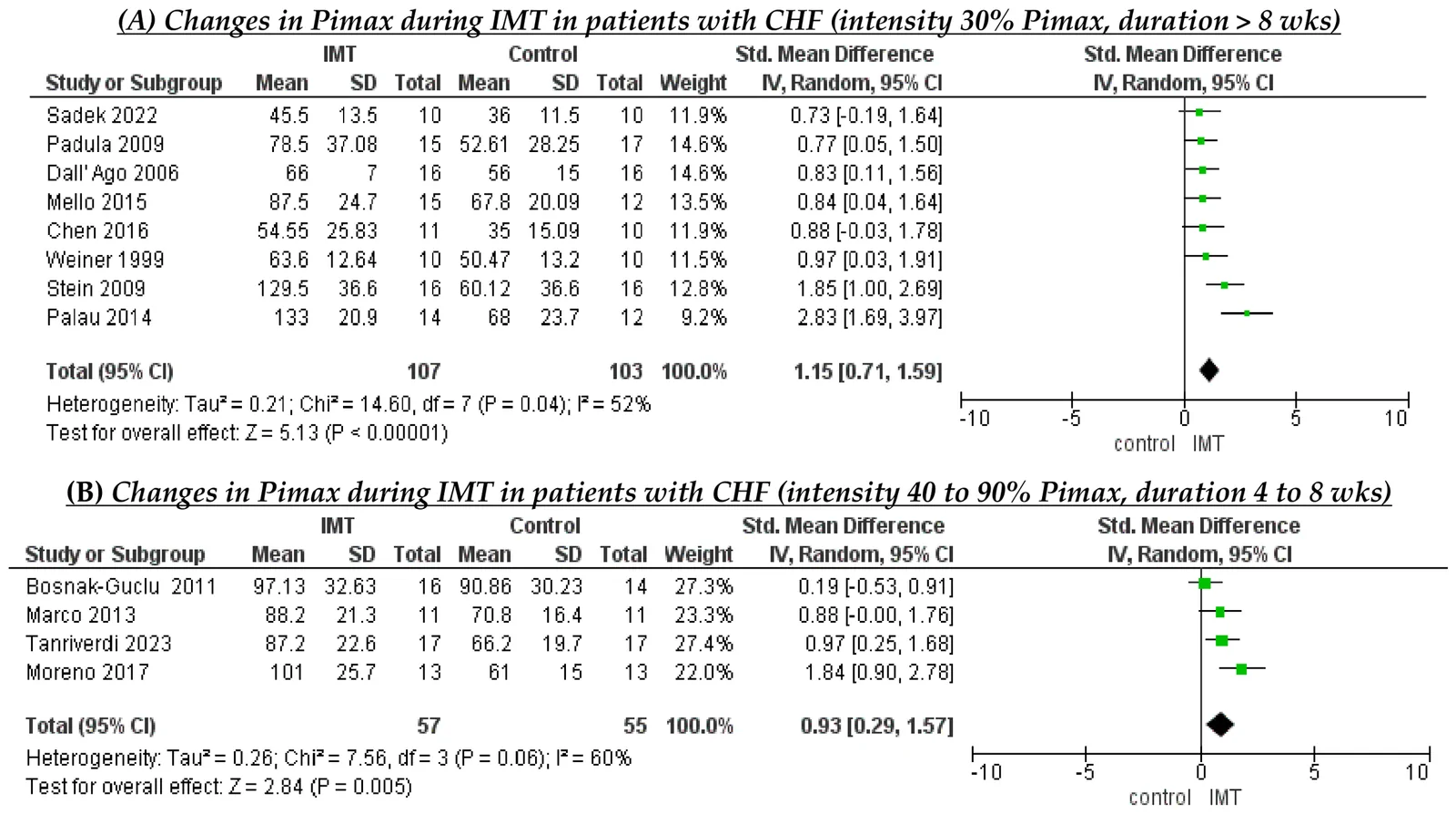
Changes in Pimax during IMT based on duration and intensity in patients with chronic heart failure [24,25,27,29,34,37,38,39,40,41,42,43].

The majority of eligible studies examined the effects of IMT on Pimax changes using training loads of 30% Pimax during a 12-week training program and found an increase in Pimax. Improvement in the patients’ maximum static Pimax during IMT seems to be attributed to changes in the diaphragm function and increased inspiratory muscle strength. Six weeks of IMT at 40% Pimax was also effective in improving Pimax [34]. Moreover, one study indicates that 8 months of IMT training using incremental resistance (30–70% Pimax) can promote inspiratory muscle strength [37]. Thus, it seems that the effect of IMT on Pimax can be provoked either with short-term high-intensity training or with long-duration training of low resistance. Similar to other skeletal muscles, respiratory muscle strength improvements are likely dose dependent. It seems that beyond the initial effect, increasing the intensity of IMT can sustain a more prolonged effect on respiratory muscle strength. Training at 30% Pimax promotes an initial training effect, but a low training load provokes a gradual decrease over time in the percentage of change. However, training at a higher load of 40 to 60% Pimax can sustain part of the effect even after four months after completion of the program. Dall Ago et al. [40] found a magnitude of improvement reaching Pimax (115%) after 12 weeks of training at 30% Pimax. However, Pimax was assessed weekly, and the training load was adjusted each week based on the updated measurements. Thus, the increased intensity during IMT seems to promote better and more prolonged effects. Sensitivity analysis performed with studies using either low intensity with long duration (Std.MD, 1,15; 95%CI, 0.71–1.59; Figure 3A, *p* < 0.00001) or moderate to high intensity with short- or medium-term duration (Std.MD, 0.93; 95%CI, 0.29–1.57; Figure 3B, *p* = 0.005) increased Pimax in patients with CHF. Thus, improvements in Pimax are attributed to total volume of training during a rehabilitation program rather than intensity or duration alone.

### 4.2. Responses of IMT to FVC

Although forced expiratory volume 1 and the ratio FEV_1_/FVC was reported in some studies, we adopted FVC assessment to examine the effect of IMT on pulmonary function because it mainly reflects lung volume physiology rather than obstructive or restrictive airflow limitation. Four studies performed over either a 12-week period at 30% Pimax or a 6-week period with high intensity reaching up to 70% Pimax improved inspiratory volumes and FVC. The increase in respiratory muscle strength, acquired by better lung mechanics and reduced congestion, may have promoted the increase in functional vital capacity.

### 4.3. Responses of IMT to Dyspnea

The critical role of IMT intensity in improving dyspnea has been outlined in the eligible studies. Short-term intervention consisting of more daily sessions at 40% Pimax led to significant differences in reducing dyspnea. Results obtained from longer intervention programs were controversial. Ten weeks of IMT at 30% Pimax performed 5 days a week did not indicate improvements to dyspnea [39], whereas in a second study [27], 12 weeks of IMT with the same intensity but higher frequency (7 days a week) improved dyspnea. The exact mechanisms for reducing dyspnea following IMT might be attributed to costal muscle strength and breathing pattern improvements [46]. In particular, IMT delays inspiratory muscle fatigue, attenuates inspiratory metaboreflex activation and reduces sympathetic vasoconstriction, leading to more efficient respiratory patterns [47]. At least one month of daily IMT is necessary to elicit beneficial results, and more beneficial results are attributed to patients with CHF with inspiratory muscle weakness than those with normal inspiratory muscle strength [38]. Studies are required to address the impact of IMT in diaphragm thickness, which will possibly benefit patients with CHF to cope with symptoms of dyspnea and pulmonary disorders. Although we found a decrease in dyspnea, whether this effect is accompanied by changes in breathing pattern and diaphragm function or whether adjustments to higher-intensity IMT are required for these changes needs to be clarified further.

### 4.4. Responses of IMT to Maximum Oxygen Uptake

A primary study reported that a low-intensity IMT protocol (25–30% Pimax) with IMT for 12 weeks did not improve VO_2peak_ [29]. In contrast, several studies showed that 12 weeks of IMT performed with high intensity reaching up to 60% Pimax was associated with a significant improvement in maximal and submaximal exercise capacity [24,38]. Similarly, high-intensity interval exercise training has been shown to elicit greater increases in VO_2peak_ compared to aerobic continuous exercise in patients with CHF [48]. Consistent with this finding, the meta-analysis highlighted the critical role of IMT intensity in VO_2peak_ changes where high-intensity IMT led to better results. Changes in VO_2peak_ were even better in patients with inspiratory muscle weakness (Pimax <70% predicted) [40]. The underlying mechanism may be that IMT enhances both sympathetic and parasympathetic regulation of the cardiovascular system and improves skeletal muscle perfusion and endothelial function, which in turn enhances respiratory function and improves oxygen metabolism during exercise in patients with CHF [38,49].

### 4.5. Applicability of Findings

Current evidence-based guidelines for patients with CHF recommend a multimodal approach incorporating aerobic, resistance, and IMT as a comprehensive exercise rehabilitation program complementary to pharmacological therapy and device application [50]. Isolated IMT may represent an adjunct approach, particularly for patients where inspiratory muscle weakness, dyspnea or ventilatory abnormalities are prominent. Thus, while isolated IMT might improve pulmonary function and metabolic responses, it should be considered preliminary and complementary to primary components of cardiac rehabilitation, such as aerobic and/or resistance training. Optimizing the integration of the exercise modalities might improve respiratory strength, exercise capacity, and clinical prognosis in patients with CHF.

Moreover, studies where frail or severely affected patients with CHF (such as those with marked respiratory muscle weakness or NYHA Class III) were included might increase heterogeneity due to differences in disease severity. Therefore, the pooled estimates should be interpreted with caution, and their applicability to specific heart failure phenotypes or patient subgroups might be limited. Nevertheless, for patients with CHF who cannot tolerate standard aerobic workloads, isolated IMT provides a convenient, low-cost intervention to improve inspiratory muscle strength. Adequate exercise volume promotes additional benefits in all outcomes. Obviously, IMT combined with aerobic training promotes additional benefits on metabolic responses [16]. Training protocols with a 10- to 12-week duration at 30% Pimax or 4 to 8 weeks of training with increased moderate to high intensity at 40 to 90% Pimax seemed to lead to desirable effects. It should be pointed out that these optimal training protocols should be considered hypotheses rather than validated methods or a substitute for guideline-based clinical decision-making. Further research is required to determine the efficacy and applicability of IMT optimal volume in clinical practice.

### 4.6. Limitations

There are certain limitations to our meta-analysis, such as the limited number of RCTs, and in most of them, the relatively small sample sizes, resulting in a modest overall sample size. The observed heterogeneity may also be attributable to variations in equipment, training frequency, intensity, duration and intervention period in the included studies. A potential source of heterogeneity is the inclusion of different IMT modalities, including incentive-based and threshold-loading interventions. Other possible sources of error in some studies include data extraction from figures. Moreover, dyspnea was assessed with different scales, increasing heterogeneity of the results. Nonetheless, the majority of studies assessed dyspnea with the MRC scale, which is less sensitive for assessing dyspnea acquired from respiratory muscle weakness.

The FEV_1_/FVC ratio was also not examined in three out of four studies. We selected to examine FVC rather than the FEV_1_/FVC ratio. The FEV_1_/FVC ratio is primarily a diagnostic index for airflow obstruction, where FVC reflects maximal lung volume and overall ventilatory capacity, which is more closely related to the assessment of pulmonary function.

## 5. Conclusions

Inspiratory muscle training may improve respiratory muscle strength, FVC, dyspnea and exercise tolerance in patients with CHF. Isolated IMT performed either as short- and medium-term intervention with moderate to high intensity reaching up to 90% Pimax (40–90% Pimax) or a long-term program with low intensity (30% Pimax) seem to promote pulmonary and metabolic responses and improve the prognosis of patients with CHF. However, these findings should be considered preliminary rather than conclusive evidence to inform clinical practice. More studies are required to conduct a quantitative synthesis that will thoroughly examine the impact of IMT on diaphragm thickness and the physiological changes on diaphragm anatomical structures in CHF. Considering the observed pulmonary and metabolic responses, clinicians could better integrate IMT into comprehensive management strategies to cope with respiratory weakness and pulmonary dysfunction in patients with heart failure.

## Figures and Tables

**Table 1:** Characteristics of the studies included in the systematic review and meta-analysis.

Studies	Participant Characteristics	IMT Intervention		Control Group	Major Findings	
		Duration	Intensity		Intervention Group	Control Group
Weiner et al., 1999 [24]	n = 20 CHF patients, 68 ± 6.2 yrs, M/F: 18/2 IMT group: n = 10 CHF patients, 66.2 ± 4.6 yrs Control group: n = 10 CHF patients 63.8 ± 4 yrs	12 wks, 6 days/wk, 1.5 h/day	15% Pimax during the 1st wk 5% increase each week 60% Pimax during the last 2 months	0% Pimax	Pimax: bsl, 46.5 ± 4.7 vs. after IMT 63.6 ± 4.0, *p* < 0.05 Dyspnea: bsl, 1.70 ± 0.3 vs. after IMT 2.70 ± 0.4, *p* < 0.005 FVC: bsl, 3.14 ± 0.5 vs. after IMT 3.37 ± 0.2, *p* < 0.05 VO_2peak_: bsl, 13.1 ± 0.8 vs. after IMT, 13.8 ± 0.4. ns mean ± SEM	Pimax: bsl, 50.7 ± 4.2 vs. after 12 wks 50.47 ± 5.1, ns Dyspnea: bsl, 1.75 ± 0.2 vs. after 12 wks, 1.70 ± 0.3, ns FVC: bsl, 3.23 ± 0.5 vs. after 12 weeks 3.12 ± 0.2 ns VO_2peak_: bsl, 13.5 ± 0.9 vs. after 12 wks,13.4 ± 0.7, ns mean ± SEM
Hossein- Pour et al., 2020 [36]	n = 84 CHF patients IMT group: n = 42 CHF patients, 55.97 ± 9.43 yrs, M/F: 23/19 Sham IMT: n = 42 CHF patients 57.28 ± 9.06 yrs, M/F: 21/21	6 wks, 7 days/wk, 30 min/day	40% Pimax	10% Pimax	Dyspnea: bsl,2.63 ± 0.79 vs. after IMT, 1.38 ± 0.66, *p* < 0.001 mean ± SD	Dyspnea: bsl, 2.19 ± 0.89 vs. after 6 wks, 2.28 ± 0.94, ns mean ± SD
Padula et al., 2009 [27]	n = 32 CHF patients, 56.62 ± 9.5 yrs IMT group: n = 15 CHF patients, 76 (51–89) yrs, M/F: 5/10 Control group: n = 17 CHF patients, 73 (32–95) yrs, M/F: 7/10	12 wks, 7 days/wk, 20 min/day	30% Pimax	standard educational protocol	Pimax: bsl,48.72 ± 25.69 vs. after IMT,78.5 ± 37.08, *p* = 0.0001 Dyspnea: bsl. 3 ± 0.6 vs. after IMT, 4.2 ± 0.4, *p*= 0.0001 mean ± SD	Pimax: bsl, 52.25 ± 27.32 vs. after 12 wks, 52.61 ± 28.25, ns Dyspnea bsl, 3.5 ± 0.8 vs. after 12 wks, 3.6 ± 0.7, ns mean ± SD
Dall Aggo et al., 2006 [40]	n = 32 CHF patients IMT group: n = 16 CHF patients, 58 ± 2 yrs, M/F: 10/6 P-IMT group: n = 16 CHF patients, 54 ± 3 yrs, M/F:11/5	12 wks, 7 days/wk, 30 min/day	30% Pimax	conventional rehabilitation program	Pimax: bsl, 59.5 ± 2.2 vs. after IMT, 66 ± 7, *p* = 0.0001 *p* < 0.01 VO_2peak_: bsl, 17 ± 0.6 vs. 21 ± 0.7 *p* < 0.001 mean ± SD	Pimax: bsl, 59.8 ± 2 vs. after 12 wks, 56 ±15, ns VO_2peak_: bsl, 17 ± 0.6 vs. after 12 wks, 17 ± 0.8, ns mean ± SD
Moreno et al., 2017 [41]	n = 26 CHF patients IMT group: n = 13 CHF patients, 58 ± 2 yrs, M/F: 10/6 Control group: n = 13 CHF patients, 54 ± 3 yrs, M/F: 11/5	8 wks, 6 times/wk, 30 min/day	40–90% Pimax	no training	Pimax: bsl, 60 ± 13 vs. after IMT, 101 ± 25.7, *p* < 0.001 VO_2peak_: bsl, 10.25 ± (7.7–12.8) vs. after IMT, 13.15 ± (10.6–14.6) *p* < 0.05 mean ± SD	Pimax: bsl, 60 ± 16 vs. 61 ± 15 after 8 wks, ns VO_2peak_: bsl, 9.95 ± (6.8–10.9) vs. after 12 wks, 8.95 ± (6.9–10.5), ns mean ± SD
Palau et al., 2014 [29]	n = 26 CHF patients IMT group: n = 14 CHF patients, 68 ± (60–76) yrs, M/F: 10/6 Control group: n = 12 CHF patients, 74 ± (73–77) yrs, M/F: 11/5	12 wks, 2 times/day for 20 min	25–30% Pimax (Pimax was measured every week)	no training	Pimax: bsl, 70 ± (55.7–84) vs. after IMT, 133 ± (92–190) *p* < 0.001 VO_2peak_: bsl, 10.25 ± (7.7–12.8) vs. after IMT, 13.15 ± (10.6–14.6) ns median and range	Pimax: bsl, 68 ± (60.5–88.5) vs. after 12 wks, 133 ± (92–190), ns VO_2peak_: bsl, 9.95 ± (6.8–10.9) vs. after 12 wks, 8.95 ± (6.9–10.5), ns median and range
Tanriverdi et al., 2023 [37]	n = 26 CHF patients IMT group: n = 14 CHF patients, 68 ± (60–76) yrs, M/F: 10/6 Control group: n = 12 CHF patients, 74 ± (73–77) yrs, M/F: 11/5	8 wks 3 days/wk, 20 min/day, 7 sets/3 min cycles	30–70% Pimax	unloaded training	Pimax: bsl, 51.7 ± 15 vs. after IMT, 87.2 ± 22.6, *p* = 0.0001 FVC: bsl, 74.6 ± 5.2 vs. after IMT, 79.1 ± 14.3, *p* = 0.004 Diaphragm thickness: bsl, 2.1 ± 0.6 vs. after IMT, 2.5 ± 0.8, ns mean ± SD	Pimax: bsl, 55.2 ±13.5 vs. after 8 wks, 66.2 ± 19.7 ns FVC: bsl, 75.2 ± 11.4 vs. after 8 wks, 77.3 ± 11.3, ns Diaphragm thickness: bsl, 2.3 ± 0.5 vs. after 8 wks, 2.5 ± 0.7, ns mean ± SD
Chen et al., 2016 [39]	n = 21 CHF patients, 65.52 ± 12.61 yrs, M/F 8/13 IMT group: n = 11 CHF patients, 63.73 ± 14.64 yrs, M/F: 4/7 Control group: n = 10 CHF patients, 67.5 ± 10.35 10.35 yrs, M/F: 4/6	10 wks, 5 days/wk, 30 min/day	30% Pimax (increase of 2 cmH_2_O/wk)	conventional rehabilitation program	Pimax: bsl, 33.64 ± 15.67 vs. after IMT, 54.55 ± 25.83, *p* = 0.008 Dyspnea: bsl, 1.45 ± 0.52 vs.after IMT, 1.55 ± 0.82, ns FVC: bsl, 2.14 ± 0.88 vs. after IMT 2.3 ± 1.01 *p* < 0.05 mean ± SD	Pimax: bsl, 44.00 ± 20.66) vs. after 10 wks, 35.00 ± 15.09 ns Dyspnea: bsl, 1.10 ± 0.32 vs.after 10 wks, 1.00 ± 0.00, ns FVC: bsl, 1.72 ± 0.55 vs. after 10 wks, 1.67 ± 0.47 ns mean ± SD
Bosnak-Guclu et al., 2011 [34]	n = 30 CHF patients IMT group: n = 16 CHF patients, 69.5 ± 7.56 yrs, M/F: 12/4 Sham IMT: n = 14 CHF patients, 65.71 ± 10.52 yrs, M/F: 12/2	6 wks, 7 days/wk, 30 min/day	40% Pimax	15% Pimax	Pimax: bsl, 62.00 ± 33.57 vs. after IMT, 97.13 ± 32.63, *p* < 0.01 Dyspnea: bsl, 2.27 ± 0.88 vs. after IMT, 1.07 ± 0.79 *p* < 0.001 FVC: bsl, 84.57 ± 15.99 vs. after IMT, 89.57 ± 14.55 *p* = 0.024 mean ± SD	Pimax: bsl, 78.64 ± 35.95 vs. after 6 wks, 90.86 ± 30.23, Dyspnea: bsl, 1.93 ±0.92 vs. after 6 wks, 1.71 ± 0.024, ns FVC: bsl, 86.75 ± 20.75 vs. 6 wks, 89.66 ± 19.97, ns mean ± SD
Marco et al., 2013 [42]	n = 22 CHF patients, 69.3± 9.66 yrs, M/F: 17/5 Training group: n = 11 CHF patients, 68.5 ± 8.88 yrs, M/F: 7/4 Sham IMT: n = 11 CHF patients, 70.1 ± 10.75 yrs, M/F: 10/1	4 wks, 7 days/wk, 2 times/day, 5 sets of 10 repetitions followed by 1–2 min of unloaded recovery breathing	10 consecutive repetitions maximum	10 cmH_2_O increased by 2.5 cmH_2_O every wk	Pimax: bsl, 56.1 ± 19.9 vs. after IMT, 88.2 ± 21.3 *p* < 0.01 Dyspnea: bsl, 2.1 ± 1.04 Vs. after IMT, 1.3 + 0.65, ns mean ± SD	Pimax: bsl, 56.1 ± 15.6 vs. after 4 wks 70.8 + 16.4 ns Dyspnea: bsl, 1.6 ± 1.03 Vs. after 4 wks, 1.3 ± 0.79 ns mean ± SD
Mello et al., 2015 [25]	n = 27 CHF patients IMT group: n = 15 CHF patients, 53.3 ± 2 yrs, M/F: 9/6 Control group: n = 12 CHF patients, 54.3 ± 2 yrs M/F: 5/7	12 wks, 7 days/wk, 3 times/day, 10 min/session	30% Pimax	0% Pimax	Pimax: bsl, 59.2 ± 4.9 vs after IMT, 87.5 ± 6.5, *p* < 0.01 VO_2peak_: bsl, 14.4 ± 0.7 vs after IMT, 18.9 ± 0.8, *p* = 0.02 mean ± SE	Pimax: bsl, 62.5 ± 5.3 vs. after 12 wks 67.8 ± 5.8, ns Sham IMT: bsl, 1.6 ± 1.03 vs. after 12 wks, 1.3 ± 0.79 VO_2peak_: bsl, 16.2 ± 0.5 vs. after 12 wks, 16.3 ± 0.6, ns mean ± SE
Sadek et al., 2022 [38]	n = 18 CHF patients, 69.3 ± 9.66 yrs IMT group: n = 11 CHF patients, 52.5 ± 13.7 yrs, M/F: NA Control group: n = 11 CHF patients, 63.4 ± 4.5 yrs M/F: NA	12 wks, 3 times/wk, 20 min/session	60% Pimax	no training	Pimax: bsl, 36.4 ± 14.3 vs. after IMT, 45.5 ± 13.5, *p* < 0.01 VO_2peak_: bsl, 8 ± 2.9 vs. after IMT, 9.7 ± 2.2, *p* = 0.02 mean ± SD	Pimax: bsl, 35.8 ± 10.5 vs. after IMT, 36 ± 11.5, ns VO_2peak_: bsl, 8.1 ± 2.1 vs. after 7.7 ± 2.2, ns mean ± SD
Stein et al., 2009 [43]	n = 32 CHF patients, 69.3 ± 9.66 yrs IMT group: n = 16 CHF patients, 52.5 ± 13.7 yrs, M/F: NA P-IMT group: n = 16 CHF patients, 63.4 ± 4.5 yrs M/F: NA	12 wks, 7 times/wk, 30 min/day	30% Pimax	without inspiratory load	Pimax: bsl, 5.9 ± 0.9 vs. after IMT, 12.7 ± 0.9 kPa; *p* = 0.001) mean ± SD	Pimax: bsl, 1428 ± 626 to 1597 ± 615 mL min_1 O2/L min_1 of VE; ns mean ± SD

CHF: chronic heart failure; FVC: functional vital capacity; IMT: inspiratory muscle training; M/F: male/female; Pimax: maximum inspiratory pressure; P-IMT: placebo inspiratory muscle training; SD: standard deviation; SEM: standard error of the mean; wks: weeks; yrs: years.

**Table 2:** Risk-of-bias assessment for randomized controlled trials.

First Author	Random Allocation Sequence Concealment Generation		Blinding of Participants & Researchers	Blinding of Outcome Assessment	Incomplete Outcome Data	Selective Reporting	Other Bias
	Selection		Performance	Detection	Attrition	Reporting	Other
Weiner, 1999 [24]	**?**	**−**	**+**	**?**	**+**	**+**	**−**
Hossein-Pour, 2020 [36]	**?**	**+**	**+**	**+**	**+**	**+**	**+**
Padula, 2009 [27]	**?**	**+**	**?**	**+**	**+**	**+**	**−**
Dall Aggo, 2006 [40]	**?**	**+**	**+**	**?**	**+**	**+**	**+**
Moreno, 2017 [41]	**+**	**?**	**+**	**+**	**+**	**+**	**+**
Palau, 2014 [29]	**?**	**+**	**+**	**?**	**+**	**+**	**+**
Tanriverdi, 2023 [37]	**+**	**?**	**+**	**+**	**+**	**+**	**+**
Chen, 2016 [39]	**?**	**?**	**?**	**?**	**+**	**−**	**+**
Bosnak-Guclu, 2011 [34]	**?**	**+**	**+**	**+**	**+**	**+**	**+**
Marco, 2013 [42]	**+**	**+**	**+**	**+**	**+**	**+**	**+**
Mello, 2015 [25]	**?**	**−**	**+**	**?**	**+**	**+**	**−**
Sadek et al., 2022 [38]	**+**	**?**	**+**	**−**	**+**	**+**	**?**
Stein, 2009 [43]	**+**	**+**	**+**	**+**	**+**	**+**	**−**

Key: **+**: low risk of bias (green); **−**: high risk of bias (red); **?**: unclear risk of bias (yellow).

**Table 3:** **CONSORT checklist:** Score represents the number of items (with percentage of items) on the checklist that were reported satisfactorily in each study.

	First Author	Design	Score
**1**	Weiner, 1999 [24]	RCT	(14.5/25) 58%
**2**	Hossein Pour, 2020 [36]	RCT	(22/25) 85%
**3**	Padula, 2009 [27]	RCT	(18/25) 72%
**4**	Dall Aggo, 2006 [40]	RCT	(18/25) 72%
**5**	Moreno, 2017 [41]	RCT	(22/25) 85%
**6**	Palau, 2014 [29]	RCT	(16.5/25) 66%
**7**	Tanriverdi, 2023 [37]	RCT	(16.5/25) 66%
**8**	Chen, 2016 [39]	RCT	(10.5/25) 42%
**9**	Bosnak-Guclu, 2011 [34]	RCT	(22/25) 85%
**10**	Marco, 2013 [42]	RCT	(25/25) 100%
**11**	Palau, 2014 [25]	RCT	(14.5/25) 58%
**12**	Sadek et al., 2022 [38]	RCT	(14.5/25) 58%
**13**	Stein, 2009 [43]	RCT	(18/25) 72%

CONSORT: Consolidated Standards of Reporting Trials; RCT: randomized controlled trial.

**Table 4:** GRADE summary of findings.

Outcomes	No. of Participants (Studies)	Quality of Evidence (GRADE)	Relative Effect (95% CI)
***Responses in patients with CHF during IMT***			
**Pimax**	324 (12)	⨁⨁⨁⨁ HIGH	Std. MD: 1.07, 95%CI: 0.72–1.42
**FVC**	110 (4)	⨁⨁⨁⨁ HIGH	Std. MD: 0.41 95%CI: 0.03–0.79
**Dyspnea**	216 (5)	⨁⨁⨁◯ MODERATE due to inconsistency	Std. MD: 1.11, 95%CI: 0.57–1.66
**VO_2peak_**	153 (6)	⨁⨁⨁◯ MODERATE due to inconsistency	Std. MD: 0.86, 95%CI: 0.06–1.66

GRADE Working group grades of evidence. ⨁⨁⨁⨁ High quality: Further research is very unlikely to change our confidence in the estimate of the effect. ⨁⨁⨁◯ Moderate quality: Further research is likely to have an important impact on our confidence in the estimate of the effect and may change the estimate. Low quality Further research is likely to have an important impact on our confidence in the estimate of the effect and is likely to change the estimate.

## Data Availability

No new data were created or analyzed in this study. Data sharing is not applicable to this article.

## Supplementary Materials

The following supporting information can be downloaded at https://www.mdpi.com/article/10.3390/healthcare14172692/s1, Figure S1. Assessment of the risk of bias for randomized controlled trials.

## References

[1] Bragazzi N.L., Zhong W., Shu J., Abu Much A., Lotan D., Grupper A., Younis A., Dai H. (2021). Burden of Heart Failure and Underlying Causes in 195 Countries and Territories from 1990 to 2017. Eur. J. Prev. Cardiol..

[2] Tomasoni D., Adamo M., Lombardi C.M., Metra M. (2019). Highlights in Heart Failure. ESC Heart Fail..

[3] Poole D.C., Richardson R.S., Haykowsky M.J., Hirai D.M., Musch T.I. (2018). Exercise limitations in heart failure with reduced and preserved ejection fraction. J. Appl. Physiol..

[4] Ziaeian F., Fonarow S. (2016). Epidemiology and Aetiology of Heart Failure. Nat. Rev. Cardiol..

[5] Kasahara Y., Izawa K.P., Watanabe S., Osada N., Omiya K. (2015). The Relation of Respiratory Muscle Strength to Disease Severity and Abnormal Ventilation During Exercise in Chronic Heart Failure Patients. Res. Cardiovasc. Med..

[6] Del Buono M.G., Arena R., Borlaug B.A., Carbone S., Canada J.M., Kirkman D.L., Garten R., Rodriguez-Miguelez P., Guazzi M., Lavie C.J. (2019). Exercise Intolerance in Patients with Heart Failure: JACC State-of-The-Art Review. J. Am. Coll. Cardiol..

[7] Ahmad S., Isbatan A., Chen S., Dudek S.M., Minshall R.D., Chen J. (2024). The Interplay of Heart Failure and Lung Disease: Clinical Correlations, Mechanisms, and Therapeutic Implications. J. Respir. Biol. Transl. Med..

[8] Hamazaki N., Kamiya K., Yamamoto S., Nozaki K., Ichikawa T., Matsuzawa R., Tanaka S., Nakamura T., Yamashita M., Maekawa E. (2020). Changes in Respiratory Muscle Strength Following Cardiac Rehabilitation for Prognosis in Patients with Heart Failure. J. Clin. Med..

[9] Rondinel Z.T., Bocchi L., Cipriano J.G., Chiappa G.R.D.S., Martins G.S., Mateus S.R.M., Cahalin L.P., Cipriano G.F.B. (2024). Diaphragm Thickness and Mobility Elicited by Two Different Modalities of Inspiratory Muscle Loading in Heart Failure Participants: A Randomized Crossover Study. PLoS ONE.

[10] Bozkurt B., Fonarow G.C., Goldberg L.R., Guglin M., Josephson R.A., Forman D.E., Lin G., Lindenfeld J., O’Connor C., Panjrath G. (2021). Cardiac Rehabilitation for Patients with Heart Failure: JACC Expert Panel. J. Am. Coll. Cardiol..

[11] Mirzai S., Sandesara U., Haykowsky M.J., Brubaker P.H., Kitzman D.W., Peters A.E. (2025). Aerobic, Resistance, and Specialized Exercise Training in Heart Failure with Preserved Ejection Fraction: A State-of-The-Art Review. Heart Fail. Rev..

[12] Torres-Castro R., Caicedo-Trujillo S., Gimeno-Santos E., Gutiérrez-Arias R., Alsina-Restoy X., Vasconcello-Castillo L., Seron P., Spruit M.A., Blanco I., Vilaró J. (2025). Effectiveness of inspiratory muscle training in patients with a chronic respiratory disease: An overview of systematic reviews. Front. Sports Act. Living.

[13] Pehlivan E., Çetinkay E., Özcan Z.B., Karaahmetoğlu F.S., Çörtük M., Ataç A., Çınarka H. (2025). Investigation of Inspiratory Muscle Training Efficiency Before Bronchoscopic Lung Volume Reduction: A Randomized Controlled Trial. Arch. Bronconeumol..

[14] Farghaly A., Fitzsimons D., Bradley J., Sedhom M., Atef H. (2022). The Need for Breathing Training Techniques: The Elephant in the Heart Failure Cardiac Rehabilitation Room: A Randomized Controlled Trial. Int. J. Environ. Res. Public Health.

[15] Ammous O., Feki W., Lotfi T., Gosselink R., Rebai A., Kammoun S. (2023). Inspiratory Muscle Training in People with Chronic Obstructive Pulmonary Disease (COPD): A Systematic Review. Cochrane Database Syst. Rev..

[16] Winkelmann E.R., Chiappa G.R., Lima C.O., Viecili P.R., Stein R., Ribeiro J.P. (2009). Addition of Inspiratory Muscle Training to Aerobic Training Improves Cardiorespiratory Responses to Exercise in Patients with Heart Failure and Inspiratory Muscle Weakness. Am. Heart J..

[17] Vibarel N., Hayot M., Ledermann B., Pellenk P.M., Ramonatxo M., Prefaut C. (2002). Effect of Aerobic Exercise Training on Inspiratory Muscle Performance and Dyspnea in Patients with Chronic Heart Failure. Eur. J. Heart Fail..

[18] Azambuja A.C.M., de Oliveira L.Z., Sbruzzi G. (2020). Inspiratory Muscle Training in Patients with Heart Failure: What Is New? Systematic Review and Meta-Analysis. Phys. Ther..

[19] Wu J., Kuang L., Fu L. (2018). Effects of Inspiratory Muscle Training in Chronic Heart Failure Patients: A Systematic Review and Meta-Analysis. Congenit. Heart Dis..

[20] Johnson P.H., Cowley A.J., Kinnear W.J. (1998). A Randomized Controlled Trial of Inspiratory Muscle Training in Stable Chronic Heart Failure. Eur. Heart J..

[21] Mancini D.M., Henson D., La Manca J., Donchez L., Levine S. (1995). Benefit of Selective Respiratory Muscle Training on Exercise Capacity in Patients with Chronic Congestive Heart Failure. Circulation.

[22] Liberati A., Altman D.G., Tetzlaff J., Mulrow C., Gřtzsche P.C., Ioannidis J.P., Clarke M., Devereaux P.J., Kleijnen J., Moher D. (2009). The PRISMA Statement for Reporting Systematic Reviews and Meta-Analyses of Studies That Evaluate HealthcareInterventions: Explanation and Elaboration. BMJ.

[23] Paiva D.N., Assmann L.B., Bordin D.F., Gass R., Jost R.T., Bernardo-Filho M., França R.A., Cardoso D.M.R. (2015). Inspiratory Muscle Training with Threshold or Incentive Spirometry: Which is the Most Effective?. Rev. Port. Pneumol..

[24] Weiner P., Waizman J., Magadle R., Berar-Yanay N., Pelled B. (1999). The Effect of Specific Inspiratory Muscle Training on the Sensation of Dyspnea and Exercise Tolerance in Patients with Congestive Heart Failure. Clin. Cardiol..

[25] Mello P.R., Guerra G.M., Borile S., Rondon M.U., Alves M.J., Negrăo C.E., Dal Lago P., Mostarda C., Irigoyen M.C., Consolim-Colombo F.M. (2012). Inspiratory Muscle Training Reduces Sympathetic Nervous Activity and Improves Inspiratory Muscle Weakness and Quality of Life in Patients with Chronic Heart Failure: A Clinical Trial. J. Cardiopulm. Rehabil. Prev..

[26] Higgins J.P.T., Thomas J., Chandler J., Cumpston M., Li T., Page M.J., Welch V.A. (2022). Cochrane Handbook for Systematic Reviews of Interventions.

[27] Padula C.A., Yeaw E., Mistry S. (2009). A Home-Based Nurse-Coached Inspiratory Muscle Training Intervention in Heart Failure. Appl. Nurs. Res..

[28] Burba B.U., O’ Connor E.A., Webber E.M., Redmond N., Perdue L.A. (2017). Estimating Data from Figures with a Web Based Program: Considerations for a Systematic Review. Res. Synth. Methods.

[29] Palau P., Domínguez E., Núñez E., Schmid J.P., Vergara P., Ramón J.M., Mascarell B., Sanchis J., Chorro F.J., Núñez J. (2014). Effects of Inspiratory Muscle Training in Patients with Heart Failure with Preserved Ejection Fraction. Eur. J. Prev. Cardiol..

[30] Wan X., Wang W., Liu J., Tong T. (2014). Estimating the Sample Mean and Standard Deviation from the Sample Size, Median, Range and/or Interquartile Range. BMC Med. Res. Methodol..

[31] The Nordic Cochrane Centre (2014). Review Manager (RevMan).

[32] Chen B., Benedetti A. (2017). Quantifying Heterogeneity in Individual Participant Data Meta-Analysis with Binary Outcome. Syst. Rev..

[33] Higgins J.P., Green S. (2011). Cochrane Handbook for Systematic Reviews of Interventions.

[34] Bosnak-Guclu M., Arikan H., Savci S., Inal-Ince D., Tulumen E., Aytemir K., Tokgözoglu L. (2011). Effects of Inspiratory Muscle Training in Patients with Heart Failure. Respir. Med..

[35] Schünemann H., Brozek J., Guyatt G., Oxman A. (2013). Handbook for Grading the Quality of Evidence and the Strength of Recommendations Using the GRADE Approach. https://gdt.gradepro.org/app/handbook/handbook.html.

[36] Hossein-Pour A.H., Gholami M., Saki M., Birjandi M. (2020). The Effect of Inspiratory Muscle Training on Fatigue and Dyspnea in Patients with Heart Failure: A Randomized, Controlled Trial. Jpn. J. Nurs. Sci..

[37] Tanriverdi A., Savci S., Ozcan Kahraman B., Odaman H., Ozpelit E., Senturk B., Ozsoy I., Baran A., Akdeniz B., Acar S. (2023). Effects of High Intensity Interval-Based Inspiratory Muscle Training in Patients with Heart Failure: A Single-Blind Randomized Controlled Trial. Heart Lung.

[38] Sadek Z., Salami A., Youness M., Awada C., Hamade M., Joumaa W.H., Ramadan W., Ahmaidi S.A. (2022). Randomized Controlled Trial of High-Intensity Interval Training and Inspiratory Muscle Training for Chronic Heart Failure Patients with Inspiratory Muscle Weakness. Chronic Illn..

[39] Chen P.C., Liaw M.Y., Wang L.Y., Tsai Y.C., Hsin Y.J., Chen Y.C., Chen S.M., Lin M.C. (2016). Inspiratory Muscle Training in Stroke Patients with Congestive Heart Failure: A Consort-Compliant Prospective Randomized Single-Blind Controlled Trial. Medicine.

[40] Dall Aggo P., Chiappa G.R., Guths H., Stein R., Ribeiro J.P. (2006). Inspiratory Muscle Training in Patients with Heart Failure and Inspiratory Muscle Weakness: A Randomized Trial. J. Am. Coll. Cardiol..

[41] Moreno A.M., Toledo-Arruda A.C., Lima J.S., Duarte C.S., Villacorta H., Nóbrega A.C.L. (2017). Inspiratory Muscle Training Improves Intercostal and Forearm Muscle Oxygenation in Patients with Chronic Heart Failure: Evidence of the Origin of the Respiratory Metaboreflex. J. Card. Fail..

[42] Marco E., Ramírez-Sarmiento A.L., Coloma A., Sartor M., Comin-Colet J., Vila J., Enjuanes C., Bruguera J., Escalada F., Gea J. (2013). High-Intensity vs. Sham Inspiratory Muscle Training in Patients with Chronic Heart Failure: A Prospective Randomized Trial. Eur. J. Heart Fail..

[43] Stein R., Chiappa G.R., Güths H., Dall’Ago P., Ribeiro J.P. (2009). Inspiratory Muscle Training Improves Oxygen Uptake Efficiency Slope in Patients with Chronic Heart Failure. J. Cardiopulm. Rehabil. Prev..

[44] Schulz K.F., Altman D.G., Moher D., CONSORT Group (2010). CONSORT 2010 Statement: Updated Guidelines for Reporting Parallel Group Randomized Trials. J. Pharmacol. Pharmacother..

[45] Chiappa G.R., Roseguini B.T., Vieira P.J., Alves C.N., Tavares A., Winkelmann E.R., Ferlin E.L., Stein R., Ribeiro J.P. (2008). Inspiratory Muscle Training Improves Blood Flow to Resting and Exercising Limbs in Patients with Chronic Heart Failure. J. Am. Coll. Cardiol..

[46] Langer D., Ciavaglia C., Faisal A., Webb K.A., Neder J.A., Gosselink R., Dacha S., Topalovic M., Ivanova A., O’Donnell D.E. (2018). Inspiratory Muscle Training Reduces Diaphragm Activation and Dyspnea During Exercise in COPD. J. Appl. Physiol..

[47] Chan J.S., Mann L.M., Doherty C.J., Angus S.A., Thompson B.P., Devries M.C., Hughson R.L., Dominelli P.B. (2023). The Effect of Inspiratory Muscle Training and Detraining on the Respiratory Metaboreflex. Exp. Physiol..

[48] Wisløff U., Střylen A., Loennechen J.P., Bruvold M., Rognmo Ø., Haram P.M., Tjønna A.E., Helgerud J., Slørdahl S.A., Lee S.J. (2007). Superior Cardiovascular Effect of Aerobic Interval Training Versus Moderate Continuous Training in Heart Failure Patients: A Randomized Study. Circulation.

[49] Cahalin L.P., Arena R.A. (2015). Breathing Exercises and Inspiratory Muscle Training in Heart Failure. Heart Fail. Clin..

[50] Taylor J.L., Myers J., Bonikowske A.R. (2023). Practical Guidelines for Exercise Prescription in Patients with Chronic Heart Failure. Heart Fail. Rev..

