# Supplementary material for: Pulmonary Function Responses During Inspiratory Muscle Training in Patients with Chronic Heart Failure: A Systematic Review and Meta-Analysis of Randomized Controlled Trials
Source: Healthcare (Basel). 2026 Aug 24;14(17):2692. doi: 10.3390/healthcare14172692 (PMC13564598; doi:10.3390/healthcare14172692)
Supplement: Supplementary file 1 [file healthcare-14-02692-s001.zip › healthcare-4460768-supplementary.pdf]

Search strategy

23,#22 NOT #21,,,"((""breathing exercise""[Title/Abstract] OR ""respiratory exercise""[Title/Abstract] OR ""pulmonary rehabilitation""[Title/Abstract] OR ""inspiratory muscle training""[Title/Abstract]) AND (""heart failure""[Title/Abstract] OR ""chronic heart failure""[Title/Abstract] OR ""congestive heart failure""[Title/Abstract] OR ""preserved heart failure""[Title/Abstract] OR ""cardiovascular disease""[Title/Abstract]) AND (((((""maximum inspiratory pressure""[Title/Abstract] OR ""diaphragm thickness""[Title/Abstract] OR ""Dyspnea""[Title/Abstract]) AND ""0R""[All Fields]) AND ""functional vital capacity""[Title/Abstract]) AND ""maximum oxygen uptake""[Title/Abstract]) OR ""pulmonary function""[Title/Abstract])) NOT (""Animals""[Title/Abstract] OR ""Children""[Title/Abstract])",22,11:18:19,2026/07/17
22,#5 AND #11 AND #18,,,"(""breathing exercise""[Title/Abstract] OR ""respiratory exercise""[Title/Abstract] OR ""pulmonary rehabilitation""[Title/Abstract] OR ""inspiratory muscle training""[Title/Abstract]) AND (""heart failure""[Title/Abstract] OR ""chronic heart failure""[Title/Abstract] OR ""congestive heart failure""[Title/Abstract] OR ""preserved heart failure""[Title/Abstract] OR ""cardiovascular disease""[Title/Abstract]) AND (((((""maximum inspiratory pressure""[Title/Abstract] OR ""diaphragm thickness""[Title/Abstract] OR ""Dyspnea""[Title/Abstract]) AND ""0R""[All Fields]) AND ""functional vital capacity""[Title/Abstract]) AND ""maximum oxygen uptake""[Title/Abstract]) OR ""pulmonary function""[Title/Abstract])",22,11:16:39,2026/07/17
21,#19 OR #20,,,"""Animals""[Title/Abstract] OR ""Children""[Title/Abstract]","2,315,400",11:15:31,2026/07/17
20,Children[Title/Abstract],,,"""Children""[Title/Abstract]","1,459,372",11:14:27,2026/07/17
19,Animals[Title/Abstract],,,"""Animals""[Title/Abstract]","864,847",11:14:14,2026/07/17
18,#12 OR #13 OR #14 0R #15 #16 OR #17,,,"((((""maximum inspiratory pressure""[Title/Abstract] OR ""diaphragm thickness""[Title/Abstract] OR ""Dyspnea""[Title/Abstract]) AND ""0R""[All Fields]) AND ""functional vital capacity""[Title/Abstract]) AND ""maximum oxygen uptake""[Title/Abstract]) OR ""pulmonary function""[Title/Abstract]","42,610",11:02:14,2026/07/17
17,Pulmonary function[Title/Abstract],,,"""pulmonary function""[Title/Abstract]","42,610",11:00:39,2026/07/17
16,Maximum oxygen uptake[Title/Abstract],,,"""maximum oxygen uptake""[Title/Abstract]","1,685",10:59:01,2026/07/17
15,Functional vital capacity[Title/Abstract],,,"""functional vital capacity""[Title/Abstract]",65,10:58:26,2026/07/17
14,Dyspnea[Title/Abstract],,,"""Dyspnea""[Title/Abstract]","57,483",10:57:31,2026/07/17
13,Diaphragm thickness[Title/Abstract],,,"""diaphragm thickness""[Title/Abstract]",473,10:57:10,2026/07/17
12,Maximum inspiratory pressure[Title/Abstract],,,"""maximum inspiratory pressure""[Title/Abstract]",745,10:56:37,2026/07/17
11,#6 OR #7 OR #8 OR #9 OR #10,,,"""heart failure""[Title/Abstract] OR ""chronic heart failure""[Title/Abstract] OR ""congestive heart failure""[Title/Abstract] OR ""preserved heart failure""[Title/Abstract] OR ""cardiovascular disease""[Title/Abstract]","469,129",10:48:11,2026/07/17
10,Cardiovascular disease[Title/Abstract],,,"""cardiovascular disease""[Title/Abstract]","215,427",10:47:33,2026/07/17
9,Preserved heart failure[Title/Abstract],,,"""preserved heart failure""[Title/Abstract]",20,10:47:07,2026/07/17
8,Congestive heart failure[Title/Abstract],,,"""congestive heart failure""[Title/Abstract]","46,610",10:46:25,2026/07/17

7,Chronic heart failure[Title/Abstract],,,""""chronic heart failure""[Title/Abstract]","21,584",10:46:01,2026/07/17
6,Heart failure[Title/Abstract],,,""""heart failure""[Title/Abstract]","270,824",10:45:23,2026/07/17
5,#1 OR #2 OR #3 OR #4,,,""""breathing exercise""[Title/Abstract] OR ""respiratory exercise""[Title/Abstract] OR ""pulmonary rehabilitation""[Title/Abstract] OR ""inspiratory muscle training""[Title/Abstract]","8,586",10:44:21,2026/07/17
4,Inspiratory muscle training[Title/Abstract],,,""""inspiratory muscle training""[Title/Abstract]","1,417",10:41:47,2026/07/17
1,Breathing exercise[Title/Abstract],,,""""breathing exercise""[Title/Abstract]",631,10:40:29,2026/07/17
3,Pulmonary rehabilitation[Title/Abstract],,,""""pulmonary rehabilitation""[Title/Abstract]","6,649",10:40:06,2026/07/17
2,Respiratory exercise[Title/Abstract]

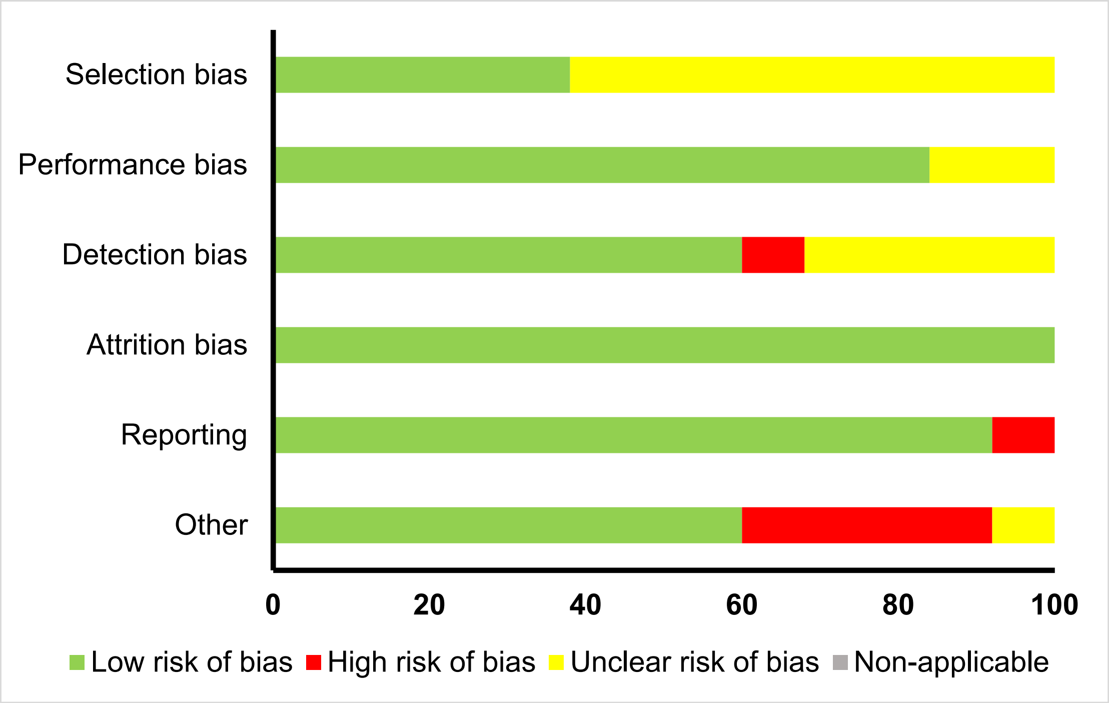

Figure S1. Assessment of the risk of bias for randomized controlled trials
